# The half-century odyssey of regulatory B cells: from Breg discovery to emerging frontiers

**DOI:** 10.3389/fimmu.2025.1681082

**Published:** 2025-09-25

**Authors:** Elina A. Zheremyan, Nikolai R. Kon, Alina S. Ustiugova, Ekaterina M. Stasevich, Elvina A. Bogomolova, Matvey M. Murashko, Aksinya N. Uvarova, Denis E. Demin, Dmitry V. Kuprash, Kirill V. Korneev

**Affiliations:** ^1^ Laboratory of Intracellular Signaling in Health and Disease, Engelhardt Institute of Molecular Biology, Russian Academy of Sciences, Moscow, Russia; ^2^ Faculty of Biology, Lomonosov Moscow State University, Moscow, Russia; ^3^ Moscow Center for Advanced Studies, Moscow, Russia

**Keywords:** regulatory B cells, Bregs, immunotherapy, immunosuppression, immunoregulation

## Abstract

For half a century, the quiet work of a specialized immunosuppressive B cell subset has been slowly unveiled, revealing its profound impact on immune balance. This review provides a comprehensive retrospective on the history of regulatory B cell (Breg) investigation, tracing their journey from initial elusive observations to their current recognition as crucial immunomodulators. We explore the paradigm shift from B cells solely as antibody producers to their multifaceted roles in immunosuppression. Key milestones include the earliest suggestions of suppressive B cell activity around 1970, the formal coining of the currently used term "regulatory B cells" in the early 2000s, and the subsequent elucidation of diverse Breg subsets and their suppressive mechanisms. Finally, we discuss contemporary advances, including the application of single-cell multi-omics, the identification of novel markers and metabolic regulators, and the promising yet challenging path toward Breg-based therapeutic strategies. This historical perspective underscores the remarkable progress in Breg biology and illuminates future directions for harnessing their clinical potential.

## 1 Introduction

B cells have long been recognized as central components of the adaptive immune system, primarily for their capacity to produce antibodies, facilitate opsonization, present antigens, and activate T cells (1). The foundational understanding of B cell biology was significantly advanced by landmark studies throughout history. For instance, the late 19th century saw Emil von Behring and Shibasaburo Kitasato identify circulating "antitoxins" (now known as antibodies) as crucial for immunity to diphtheria and tetanus (2). Paul Ehrlich later proposed that cells with pre-formed antibody receptors were the producers of these "antitoxins," laying theoretical groundwork (3). The cellular source of antibodies, B cells, was more definitively identified in the late 1940s, with plasma cell development correlating with antibody responses after immunization (4). A pivotal moment arrived in 1965 with Max Cooper and Robert Good's landmark study using chicken models, which established B cells as a distinct lineage responsible for antibody production, separate from T cells involved in delayed-type hypersensitivity (5).

**Figure 1:**
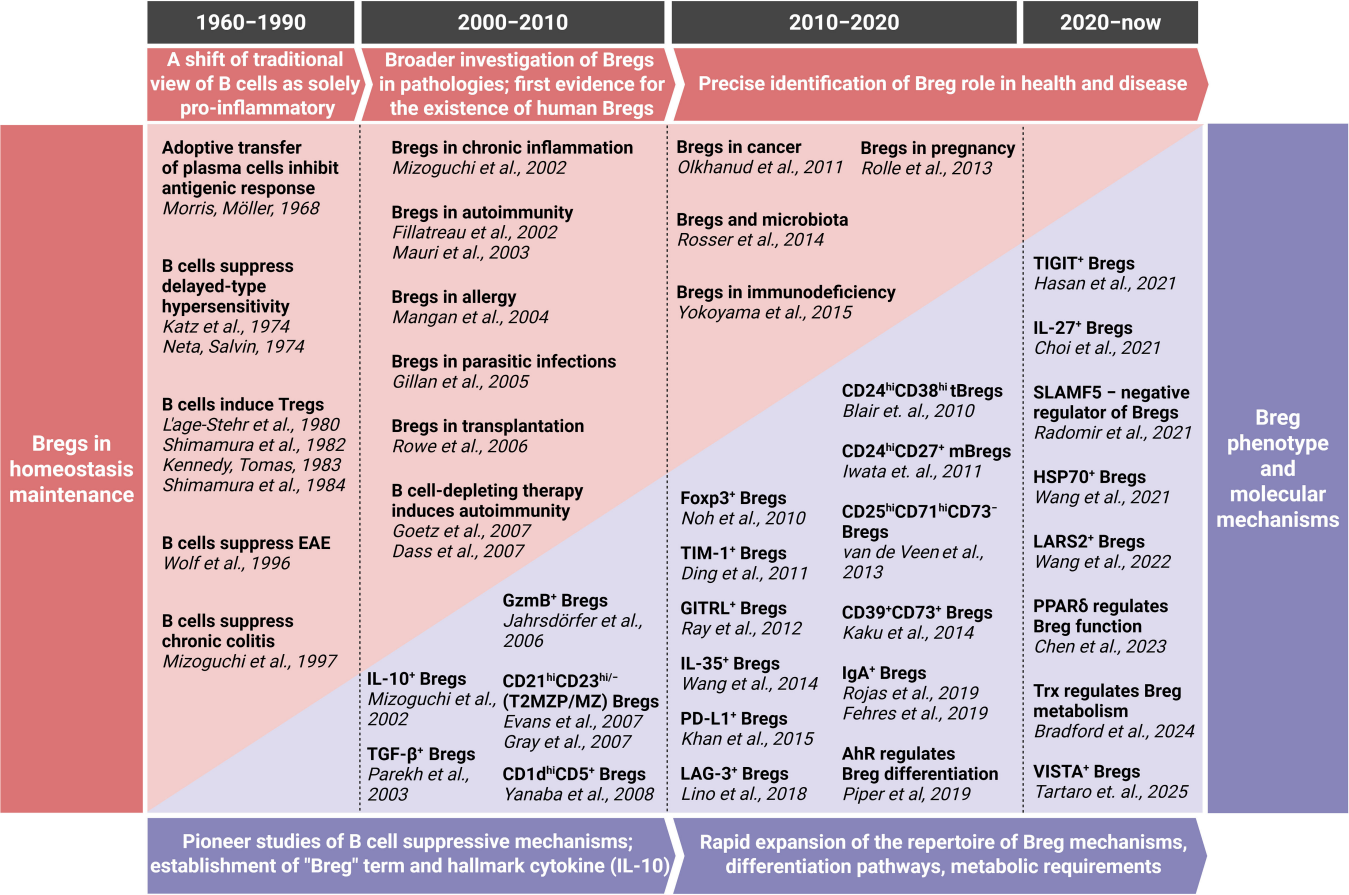
Timeline of Breg journey. For a more comprehensive overview, including additional key discoveries and detailed contextual descriptions, please refer to **Table 1**. EAE, experimental autoimmune encephalomyelitis; GzmB, granzyme B; LARS2, leucine-tRNA-synthetase-2; PPARδ, peroxisome proliferator-activated receptor delta; Trx, thioredoxin. Created with BioRender.com.

The initial discovery and characterization of B cells focused predominantly on their role as antibody producers and promoters of adaptive immunity, establishing a long-held view of their function. However, this established immunological paradigm began to shift fundamentally with the emergence of observations suggesting a suppressive capacity for B cells. The first hints of B cells possessing immunosuppressive properties emerged in the late 1960s (6) However, given the lack of understanding regarding the molecular mechanisms underlying the function of these B cells, the concept of anti-inflammatory B lymphocytes faced significant challenges in gaining widespread acceptance within the scientific community. The currently used well-established term "regulatory B cells" (Bregs) itself emerged relatively recently after the publication of data on the immunosuppressive role of B lymphocytes in chronic intestinal inflammation (7). Later, it has been demonstrated that Bregs play a direct role in the pathogenesis of a wide range of pathologies, including cancer, autoimmune diseases, infectious diseases, and transplantation immunity (8, 9). In addition, these cells have been shown to contribute to the maintenance of homeostasis in a healthy organism (8). Recent progress in the field has led to a significant advancement in our understanding of Breg origin, differentiation pathways and molecular mechanisms of immunosuppression. While numerous reviews have discussed the mechanisms and functional roles of Bregs, few have synthesized their scientific journey – how the concept first emerged, how acceptance within the immunology community evolved, and how successive methodological advances reshaped our understanding, from early skepticism to recognition of Bregs as key immunoregulatory players. We also highlight unresolved controversies and translational frontiers. Major milestones in the Breg field are shown in **Table 1** and represented in the timeline (**Figure 1**).

**Table 1:** Key milestones in regulatory B cell research.

Year	Key discovery	Researchers	Brief significance
1968	Adoptively transferred plasma cells inhibit subsequent response to antigenic stimulation	Morris and Möller (6)	First demonstration of B cell suppressive function, challenging the antibody-centric paradigm and laying foundation for regulatory B cell concept
1974	B cell-depleted splenocytes exacerbate delayed-type hypersensitivity in guinea pigs	Katz et al., Neta and Salvin (11, 12)	
1980-1984	Adoptive transfer of BCR-activated B cells induces tolerance and Treg differentiation in mice	L'age‐Stehr et al., Shimamura et al., Kennedy et al. (14–17)	Provided compelling *in vivo* evidence that B cells can drive immune tolerance by inducing Treg differentiation, reinforcing the emerging suppressor B cell concept
1996	*In vivo* demonstration of immunoregulatory B cells in EAE	Wolf et al. (19)	Landmark *in vivo* evidence that B cells are essential for controlling and resolving autoimmune inflammation, confirming their immunoregulatory role
	B cells express functional FasL	Hahne et al. (60)	Revealed cell death-mediated suppression as a novel mechanism of B cell immunoregulation
1997	Immunoregulatory B cells in the chronic colitis model	Mizoguchi et al. (20)	Confirmed the protective role of B cells in chronic intestinal inflammation, strengthening the case for their suppressive capacity *in vivo*
2001	B cells are essential for immunoregulation in immune privileged organs	D'Orazio et al. (61)	Demonstrated that B cells are central to maintaining immune tolerance in specialized tissues, expanding the relevance of Bregs beyond systemic immunity
2002	Formal coining of the term "regulatory B cells" (Bregs) and identification of IL-10-producing B cells in chronic inflammation	Mizoguchi et al. (7)	Provided a clear identity for suppressive B cells and established IL-10 as their hallmark cytokine, shaping the modern Breg framework
	Bregs ameliorate EAE and CIA via IL-10-dependent suppression	Fillatreau et al. (62)	Demonstrated that IL-10 production by Bregs can suppress autoimmune disease, firmly linking IL-10 to Breg function
2003	Bregs ameliorate CIA via IL-10-dependent suppression	Mauri et al. (63)	
	TGF-β^+^ B cells induce anergy in T killers	Parekh et al. (64)	Highlighted TGF-β as an alternative suppressive pathway, broadening the mechanistic repertoire of Bregs
2004	IL-10^+^ B cells protect from systemic anaphylaxis	Mangan et al. (65)	Linked Bregs to allergic tolerance, expanding their role beyond autoimmunity
2005	IL-10^+^ B cells suppress immunity to parasites	Gillan et al. (66)	Demonstrated that Bregs regulate host-pathogen interactions, revealing their relevance in infectious disease
2006	The concept of Bregs is formally established	Mizoguchi et al. (21)	Unified diverse findings under the formal “Breg” terminology, consolidating the field
	Human B cells produce GzmB in response to IL-21 and BCR stimulation	Jahrsdörfer et al. (67)	Identified cytotoxic granzyme B production as a novel suppressive mechanism, expanding functional diversity
	Bregs attenuate GvHD after bone marrow transplantation	Rowe et al. (68)	Demonstrated the therapeutic potential of Bregs in transplantation by showing their ability to ameliorate graft-versus-host disease.
2007	Anti-CD20 (rituximab) B cell depletion exacerbates ulcerative colitis in patients	Goetz et al. (23)	Provided clinical evidence that Bregs exist in humans, as their removal worsened inflammatory disease
	Rituximab-linked psoriasis onset highlights Breg protective role	Dass et al. (22)	
	Discovery of immunosuppressive CD21^hi^CD23^hi^ (T2-MZP) and CD21^hi^CD23σπ− σπ&Bregs (MZ)	Evans et al., Gray et al. (69, 70)	Identified novel murine Breg subsets, demonstrating phenotypic diversity within regulatory B cells
2008	Identification of functional IL-10^+^CD1d^hi^CD5^+^ Breg subset ("B10") in mice	Yanaba et al. (71)	
2009	Breg (B10) development requires BCR diversity and TLR signaling	Yanaba et al. (72)	Provided mechanistic insight into Breg induction, linking their development to innate and adaptive signals
2010	First identification of human Bregs (CD24^hi^CD38^hi^) in SLE	Blair et al. (26)	Established a distinct human Breg phenotype and showed their dysfunction in autoimmunity, opening human Breg research
	Foxp3^+^CD5^+^ Bregs in human PBMC	Noh et al. (73)	Suggested phenotypic overlap between Bregs and Tregs, raising questions about lineage and plasticity
2011	Tumor-associated B cells suppress antitumor immunity	Olkhanud et al. (74)	Linked Bregs to cancer immune evasion, highlighting their potential as therapeutic targets in oncology
	TIM-1 as a marker for IL-10^+^ Bregs, and its role in tolerance	Ding et al. (59)	Identified a functional marker and target for modulating Breg-mediated tolerance
2012	CD25^hi^ Bregs suppress effector T cells and induce Tregs	Kessel et al. (75)	Expanded the Breg repertoire by identifying a subset that promotes Treg induction
	Bregs maintain Tregs via GITRL expression	Ray et al. (76)	Demonstrated that Bregs sustain immune tolerance by directly supporting Treg homeostasis
2013	Bregs support fetal-maternal tolerance	Rolle et al. (77)	Linked Bregs to pregnancy immunology, suggesting their role in preventing fetal rejection
	CD25^hi^CD71^hi^CD73^−^ "Br1 cells" suppress inflammation and induce Tregs	van de Veen et al. (78)	Expanded Breg phenotypic diversity with identification of Br1 cells in humans
	Murine CD5^+^CX3CR1^+^ "TolBC" induce Tregs via TGF-β	Liu et al. (79)	Described a novel murine Breg subset, reinforcing heterogeneity of regulatory B cells
2014	CD19^+^CD27^int^CD38^+^ plasmablasts inhibit DC function	Matsumoto et al. (80)	Showed that antibody-secreting plasmablasts can also exert regulatory functions
	IL-35 as a Breg-produced cytokine suppressing immune responses	Shen et al. (81)	Expanded the cytokine repertoire of Bregs beyond IL-10 and TGF-β, underscoring mechanistic diversity
	Commensal gut microbiota induces Bregs	Rosser et al. (82)	Highlighted the role of the microbiome in shaping Breg development and function
	Adenosine production by CD39^+^CD73^+^ B cells as a suppressive mechanism	Kaku et al. (83)	Revealed metabolite-mediated immunoregulation as a novel Breg mechanism
2015	Bregs express IDO, promoting Treg proliferation	Nouël et al. (84)	Linked tryptophan metabolism to Breg function, further expanding suppressive pathways
	PD-L1-expressing B cells suppress T cells via PD-1/PD-L1 pathway	Khan et al. (85)	Connected Bregs to immune checkpoint biology, aligning them with therapeutic targets
	CD1d^hi^CD5^+^ Breg role identified in Wiskott-Aldrich Syndrome	Yokoyama et al. (86)	Showed that Breg defects contribute to immunodeficiency, linking them to orphan diseases
2018	Identification of LAG-3+ regulatory plasma cells	Lino et al. (87)	Demonstrated that plasma cells can adopt a regulatory identity, expanding Breg phenotypic diversity
2019	Commensal-reactive IgA-producing recirculating Bregs modulate neuroinflammation	Rojas et al. (32)	Highlighted IgA^+^ Bregs as a distinct subset, while APRIL serves as an inducer of this phenotype
	IgA^+^ Bregs can be induced by APRIL	Feher et al. (33)	
	AhR as a transcriptional regulator of Breg differentiation	Piper et al. (29)	Revealed environmental sensing as a key factor in Breg differentiation
2021	IL-27-producing Bregs suppress neuroinflammation	Choi et al. (42)	Identified IL-27^+^ Breg subset, expanding cytokine-mediated mechanisms of suppression
	SLAMF5 as a negative regulator of IL-10^+^ Bregs	Radomir et al. (50)	Proposed SLAMF5 as a novel therapeutic target for stabilizing Bregs
	TIGIT^+^ memory Bregs suppress T cells and DC	Hasan et al. (38)	Revealed a human Breg subset with multi-mechanism suppression, broadening functional scope
	B cell-derived GABA induces anti-inflammatory macrophages	Zhang et al. (88)	Uncovered neurotransmitter-mediated immunoregulation, linking Bregs to neural-immune interactions
	Breg-derived HSP70 suppresses CD4^+^ T cells	Wang et al. (89)	Identified stress chaperone-mediated suppression as a novel Breg function
2022	LARS2-expressing B cells promote tumor immunoevasion	Wang et al. (37)	Linked mitochondrial metabolism to tumor-promoting Breg subsets
2023	PPARδ is essential for tumor-induced IL-10^+^ Bregs, and its inhibition enhances immunotherapy	Chen et al. (51)	Demonstrated metabolic control of Bregs in cancer, offering therapeutic intervention points
2024	Breg-derived antibodies shape neonatal gut microbiome	Gu et al. (90)	Showed that neonatal Bregs influence early-life microbiota development, broadening physiological relevance
	OXPHOS-dependent IL-10^+^ Bregs; Trx deficiency in SLE Bregs	Bradford et al. (49)	Linked mitochondrial metabolism and redox balance to Breg differentiation and autoimmune dysfunction
2025	VISTA^+^ Bregs suppress immunity via VISTA–PSGL-1 axis	Tartaro et al. (40)	Identified VISTA^+^ Bregs as pro-tumor subsets, highlighting novel immunosuppressive axes

EAE, experimental autoimmune encephalomyelitis; CIA, collagen-induced arthritis; GzmB, granzyme B; GvHD, graft-versus-host disease; TolBC, tolerogenic B cells; DC, dendritic cells; IDO, indoleamine 2,3-dioxygenase; GABA, gamma-aminobutyric acid; LARS2, leucine-tRNA-synthetase-2; PPARδ, peroxisome proliferator-activated receptor delta; OXPHOS, oxidative phosphorylation; Trx, thioredoxin.

## 2 The genesis of immunosuppressive B cells (1960s – 1990s)

The journey into understanding the immunosuppressive capabilities of B cells began in the late 1960s, challenging the then-prevailing view of B lymphocytes solely as antibody-producing effector cells (10). This direct contradiction to the perceived "promoter" role of B cells necessitated a re-evaluation of their fundamental functions, introducing a new dimension to B cell functionality and indicating that B cells possess a dual potential: both pro-inflammatory and anti-inflammatory.

Morris and Möller were the first to discover that adoptively transferred plasma cells can inhibit response to antigenic stimulation (6). A significant turning point came with experiments demonstrating that B cell-depleted splenocytes exhibited a reduced capacity to suppress delayed-type hypersensitivity (DTH) reactions in guinea pigs. Specifically, studies by Katz, Parker, and Turk in 1974, and Neta and Salvin in the same year, showed that B cell depletion led to a more intense and prolonged DTH response compared to controls (11, 12). These findings directly implied that B cells could actively inhibit T cell activity, introducing the concept of a "suppressor B cell" (10). While the precise mechanisms remained unclear at that early stage, these observations were crucial in broadening the understanding of B cell functionality beyond antibody production (10). The notion that B cells could contribute to immune regulation, similar to the emerging understanding of suppressor T cells, laid the conceptual groundwork for future investigations into Breg subsets (13). Subsequent studies in the 1980s further supported this hypothesis, demonstrating that adoptive transfer of activated splenic B cells could induce tolerance and promote the differentiation of T cells into suppressor T cells (14–17). The 1990s marked a period of more definitive *in vivo* demonstrations of B cell-mediated immune suppression, largely facilitated by the development of genetically modified mouse models (10). A pivotal advancement was the establishment of a B cell-deficient mouse strain, µMT, achieved by disrupting the immunoglobulin µ chain gene (18). These mice, lacking mature B cells, became an invaluable tool for dissecting the roles of B cells in various immune contexts (10). A landmark study by Janeway and colleagues in 1996 utilized these µMT mice to investigate the role of B cells in experimental autoimmune encephalomyelitis (EAE), a widely used murine model for multiple sclerosis (19). While the incidence and initial severity of EAE were comparable between wild-type and µMT mice, the B cell-deficient mice exhibited a significantly longer disease duration and rarely achieved full recovery. This observation strongly suggested that B cells played a crucial role in the resolution or immune modulation of an acute autoimmune reaction in the central nervous system, rather than solely contributing to its pathogenesis (19). Further solidifying the concept of B cell-mediated immune suppression, similar findings were reported in a chronic colitis model in 1997 (20). Research demonstrated that mice lacking B cells developed colitis at an earlier age and experienced more severe disease compared to their B cell-sufficient counterparts (20). The adoptive transfer of purified B cells from healthy mice could prevent the development of colitis in recipient mice, indicating a protective, suppressive role for B cells in inflammatory bowel conditions.

These *in vivo* demonstrations in well-defined experimental murine models provided compelling evidence for the existence of B cells with immunoregulatory properties, setting the stage for their formal identification and characterization in the subsequent decade.

## 3 Coining the term and early characterization (2000s – 2010s)

The early 2000s marked a pivotal moment in B cell immunology with the formal coining of the term "regulatory B cells" (7). This distinct identity for the B cell subset responsible for immunosuppressive functions has moved beyond the earlier, less defined "suppressor B cell" hypothesis.

In 2006, Mizoguchi and colleagues were instrumental in introducing the term “Bregs” to the scientific community (21). Their research, conducted in mouse models of colitis and intestinal inflammation, identified a discrete population of B cells that exhibited suppressive functions (7, 20). These B cells were observed to expand in chronic inflammatory environments and were capable of dampening the progression of intestinal inflammation by down-regulating inflammatory signaling pathways. This work provided a clear conceptual framework and a specific name for this immunomodulatory B cell population.

The hypothesis of human Bregs was initially based on clinical observations. One of the first pieces of evidence came from observations related to the use of the B cell-depleting antibody rituximab. In some patients treated with rituximab, B cell depletion was associated with the development of psoriasis or a worsening of ulcerative colitis (22, 23). These paradoxical outcomes implied that B cells can exert a suppressive function in humans, which was lost upon their depletion. Further support for the human Breg hypothesis emerged when a clinical trial involving anti-CD20 monoclonal antibody therapy in transplant recipients was halted due to an increased rate of organ rejection (24). These early indications suggested that B cells also exert immunosuppressive functions in humans, similar to findings in murine models (25).

More definitive identification of human Bregs occurred in 2010 when Claudia Mauri's group identified them in the context of systemic lupus erythematosus (SLE) (26). They identified a specific immature B cell population in human peripheral blood, characterized by the CD24^hi^CD38^hi^ phenotype. These cells were shown to produce high amounts of IL-10 upon *in vitro* CD40 engagement and were capable of suppressing Th1 differentiation and converting CD4^+^ T cells into regulatory T cells (Tregs).

Following the formal coining of the term, IL-10 rapidly emerged as the hallmark suppressive cytokine associated with Breg activity in the early 2000s. Its consistent involvement in Breg-mediated immune suppression across various models established it as the primary functional molecule for many years (1). For a long time, Bregs were identified solely by the expression of IL-10. Later, the existence of IL-10-independent Bregs has been convincingly demonstrated, and the term “B10 cells” was commonly used to refer to this population. However, it has become increasingly clear that IL-10 expression alone does not fully capture the phenotypic and functional heterogeneity of Bregs.

## 4 Advances in Breg subset characterization and differentiation pathways (2010s – 2020s)

The next era of Breg research marked the discovery of a large variety of human and murine Breg subsets, including common interspecies subpopulations such as CD5^+^CD1d^+^, as well as unique ones, such as CD24^hi^CD38^hi^ (transitional Bregs), CD24^hi^CD27^+^ (memory Bregs, also termed “B10” in humans), CD25^+^CD71^+^CD73^−^ (Br1) for humans, and CD21^hi^CD23^hi^ (T2-MZP, transitional 2 marginal zone precursor B cells), CD21^hi^CD23^−^ (MZ, marginal zone B cells), CD138^+^CD44^hi^ (plasmablasts) for mice (1, 27). Breg subpopulations can also be identified based on their effector anti-inflammatory molecules, which broadly fall into three categories: cytokine-mediated mechanisms (TGF-β^+^, IL-35^+^, IL-10^+^, etc.), cell-cell contact mechanisms (PD-L1, TIM-1, FasL, TIGIT, etc.), and metabolic or unconventional mechanisms (CD39/CD73/adenosine, thioredoxin, GABA, HSP70) (28). It is noteworthy that despite this phenotypic heterogeneity some of these populations may overlap. With the discovery of a wide range of Breg subsets, scientists kept questioning the origin of Bregs. Hypotheses of pathways for Breg differentiation have long been debated. The basic concept suggested the presence of a universal Breg lineage marker (more possibly – transcription factor), and a lot of attempts have been made to find one. In 2019, Claudia Mauri’s group succeeded in finding a transcriptional factor aryl hydrocarbon receptor (AhR), which was shown to contribute to the CD21^hi^CD24^hi^ Breg differentiation (29). Another transcription factor, hypoxia-inducible factor-1α, was also found to regulate CD1d^hi^CD5^+^ Bregs. The master-regulator of Tregs, FOXP3, was also identified in a subset of Bregs (30). However, to date, no study has succeeded in identifying a universal Breg lineage marker. Taking into account the high heterogeneity of Breg subsets and the ability of B cells to acquire regulatory functions in response to specific stimuli, the concept of an inducible Breg nature has arisen. This concept has been proven in a wide variety of studies showing that Bregs can be induced from different subsets of B cells (31–33). Breg-inducing stimuli include CD40L, IL-21, IL-35, CpG, BAFF, and APRIL in different combinations (33–36). This concept of "induced Bregs" highlights the plasticity of B cells and their ability to adapt their function based on the local immune milieu, also providing a basis for their therapeutic implication.

This era marked the burst of articles in the Breg field. The majority of these publications consist of observations regarding the involvement of Bregs in the pathogenesis of various diseases, mainly different types of cancer, autoimmune diseases, infections, and graft-versus-host disease (25). It was essential to transition towards a detailed investigation of their mechanisms of immunoregulation, intensifying the study of their molecular characteristics and methods for precise manipulation of Bregs.

## 5 Omics revolution in Breg immunology (2020s – onwards)

The current era is profoundly shaped by the advent and widespread adoption of single-cell and multi-omics technologies. These high-resolution analytical techniques are deepening the understanding of Breg heterogeneity and function, providing an unprecedented level of precision that was previously unattainable with traditional methods. Traditional bulk sequencing and flow cytometry often masked the true diversity within B cell populations by providing an average view of gene expression. Single-cell RNA sequencing (scRNA-seq), single-cell B cell receptor sequencing (scBCR-seq), and integrated multi-omics approaches overcome these limitations. This capability is critical for unraveling the true diversity of Bregs, which are known to exhibit remarkable phenotypic variability depending on their tissue location and the specific disease context.

Recent research leveraging these advanced technologies has already yielded significant discoveries, such as the identification of novel Breg subsets and Breg effector mechanisms (TIGIT^+^ Bregs, LARS2^+^ Bregs, VISTA^+^ Bregs, etc.) (37–40). Single-cell analysis has recently delineated seven organ-specific Breg subsets with variable immunosuppressive functions in mice, each with distinct gene expression profiles and immunosuppressive functionalities (41). These modern techniques have also helped reveal the functional diversity of Bregs. In particular, functionally specific Bregs that minimally express IL-10 (previously considered Breg hallmark) but show high levels of TGF-β and IL-35 were characterized, which proved that IL-10^−^ Breg cells also possess specific immunosuppressive properties distinct from conventional Bregs (41). Recently, transcriptomic analysis revealed a distinct subpopulation of IL-27^+^ Bregs, which is also developmentally and functionally distinct from IL-10^+^ and IL-35^+^ Bregs (42). Notably, Bregs exhibit organ-specific heterogeneity with diverse phenotypes and functions depending on their organ of residence, such as the spleen, lymph nodes, or peritoneal cavity (41). This heterogeneity highlights the need for detailed characterization of Breg subsets across different tissues, and technologies like scRNA-seq offer a powerful approach to map their functional diversity in various contexts. Complementing omics-powered insights, recent research emphasizes that epigenetic and genetic regulation critically shape Breg functionality: histone modifications and DNA methylation, regulatory RNAs (lncRNA, miRNA, circRNA, etc.) and non-coding SNPs influence B cell subset differentiation and activation, thereby contributing to disease pathogenesis (43–48).

Another key milestone has been the refinement of our understanding of Breg differentiation and its requirements. A recent study by Mauri’s group performed trajectory analysis of scRNA-seq data of B cell culture activated with Breg-inducing CpG and revealed pathways of Breg differentiation. Within these pathways, they identified a specific metabolic regulator of Breg differentiation – a redox-regulating protein thioredoxin, highlighting that Breg differentiation, unlike non-Breg cells, highly depends on mitochondrial electron transport and controlled reactive oxygen species levels, while inhibition of other metabolic pathways made no difference (49). It is important to note that some other important regulators of Breg differentiation and function have also been recently identified, including PPARδ and SLAMF5, posing new potential therapeutic targets for Breg modulation (50, 51).

## 6 Emerging implications of Bregs

Bregs have attracted increasing attention as potential agents for immune modulation with human studies providing evidence across key contexts such as in autoimmune diseases (impaired transitional Bregs in SLE, paradoxical flares after rituximab in ulcerative colitis, etc.) (23, 26), transplantation (Breg-associated cytokines and the frequency of circulating Bregs as biomarkers of allograft rejection, implementation of Bregs to prevent graft-versus-host disease, etc.) (52–54), and cancer (VISTA^+^, PD-1^+^PD-L1^+^ and other tumor-associated Bregs as drivers of immune evasion, etc.) (40, 55). Recent advances have paved the way for several promising therapeutic approaches aimed at harnessing the immunosuppressive functions of Bregs.

While adoptive transfer of Tregs has advanced further clinically (56), with numerous trials and translational studies already underway, development of Breg-based cell therapy is still in its early stages (57). Nevertheless, Bregs offer unique advantages in some contexts at least due to their ability of antigen presentation and production of tolerogenic antibodies, providing a wider spectrum of immunoregulatory mechanisms. Adoptive Breg cell therapy represents a rapidly evolving strategy, involving the *ex vivo* expansion of Bregs and their subsequent reintroduction into the patient. Protocols have been developed to generate human Bregs from either autologous or allogeneic peripheral B cells using specific inducers. These expanded Bregs exhibit strong suppressive activity and the capacity to modulate effector cell responses, offering a foundation for personalized Breg-based immunotherapies (58). Engineering of antigen-specific Bregs also holds promise for the treatment of autoimmune pathologies characterized by involvement of well-defined autoantigens.

Given Breg ability to interact with and promote other immunoregulatory cell populations, including Tregs, myeloid-derived suppressor cells, and invariant natural killer T cells, they present an attractive option for combination immunotherapeutic strategies (1). Co-administration or co-induction of Bregs (for example, with *in vivo* inductors such as short-chain fatty acids, etc.) with other regulatory cells may enhance immune tolerance and provide longer-lasting immunosuppressive effects.

Advances in the identification of Breg-specific markers and regulatory pathways have opened new avenues for *in vivo* modulation. Even though Breg-specific lineage markers are yet to be identified, molecules such as TIM-1, TIGIT, and others have emerged as functional markers and potential therapeutic targets (38, 59). Pharmacologic agents or biologics designed to enhance or inhibit these pathways may allow for precise manipulation of Breg function in disease-specific contexts, enabling a shift toward immune tolerance without broad immunosuppression.

## 7 Conclusions and perspectives

The journey of Bregs has evolved from the initial recognition of a suppressive B cell population primarily defined by IL-10 production to a sophisticated understanding of a heterogeneous family of cells employing diverse suppressive mechanisms. The early 2000s established the fundamental concept of Bregs as critical regulators of immune homeostasis, particularly in autoimmune contexts. However, the subsequent decades, culminating in the current era, have revealed that Bregs are not a singular entity but rather a dynamic and adaptable population capable of acquiring regulatory functions through various pathways.

The current landscape, characterized by an enhanced ability to dissect cellular heterogeneity using single-cell and multi-omics technologies, has led to the identification of novel markers, subsets, and intricate regulatory networks beyond the B10 concept. The understanding that Bregs can arise from conventional B cell subsets in a context-dependent manner represents a major paradigm shift. Despite all the advances in the field, a significant challenge persists: Bregs still lack a unique lineage marker, which absence makes their identification and classification a persistent challenge; however, it remains uncertain whether such a marker exists. Beyond this, additional barriers remain, including the plasticity of Breg phenotypes, their strong context-dependency across diseases and tissues, and methodological limitations that complicate translation from experimental models to human biology. Deeper appreciation of Breg biology is not just academic: it directly informs the development of targeted therapeutic strategies. The creation of composite Breg-specific signatures with predictive potential could allow for the classification of risk groups in different Breg-associated pathologies, as well as for more precise therapy selection. Exploration of organ-specific Bregs is also critical, as distinct tissue-resident subsets may employ unique suppressive mechanisms that influence disease outcomes. Moreover, investigating combinatorial regulatory cell therapies could reveal synergistic approaches to enhance immunomodulation. Further challenges stem from pronounced heterogeneity of Bregs, as distinct subpopulations may use non-overlapping suppressive mechanisms, making it unclear which subset should be expanded or targeted therapeutically; their localization, since certain Breg subsets are scarce in peripheral blood and thus difficult to harvest for adoptive transfer; and the potential risks of Breg-based cellular products, which could cause excessive immunosuppression and predispose to malignancy. Moreover, it remains a major challenge to direct Bregs selectively to diseased tissues, in order to avoid systemic suppression and maximize their therapeutic benefit in a localized manner. A clear reflection of these barriers is the current translational gap: despite promising preclinical data, Breg-based therapies have not yet entered clinical trials. The new era of Breg immunology is poised to unlock the full therapeutic potential of these cells, offering novel avenues for treating a wide array of immune-mediated diseases and significantly advancing the field of immune tolerance.
